# Synthesis of ternary sulfide nanomaterials using dithiocarbamate complexes as single source precursors

**DOI:** 10.1039/c9na00275h

**Published:** 2019-06-18

**Authors:** Anna Roffey, Nathan Hollingsworth, Graeme Hogarth

**Affiliations:** Department of Chemistry, King's College London Britannia House, 7 Trinity Street London SE1 1DB UK graeme.Hogarth@kcl.ac.uk; Department of Chemistry, University College London 20 Gordon Street London WC1H OAJ UK

## Abstract

We report the use of cheap, readily accessible and easy to handle di-isobutyl-dithiocarbamate complexes, [M(S_2_CN^i^Bu_2_)_*n*_], as single source precursors (SSPs) to ternary sulfides of iron–nickel, iron–copper and nickel–cobalt. Varying decomposition temperature and precursor concentrations has a significant effect on both the phase and size of the nanomaterials, and in some instances meta-stable phases are accessible. Decomposition of [Fe(S_2_CN^i^Bu_2_)_3_]/[Ni(S_2_CN^i^Bu_2_)_2_] at *ca.* 210–230 °C affords metastable FeNi_2_S_4_ (violarite) nanoparticles, while at higher temperatures the thermodynamic product (Fe,Ni)_9_S_8_ (pentlandite) results. Addition of tetra-isobutyl-thiuram disulfide to the decomposition mixture can significantly affect the nature of the product at any particular temperature-concentration, being attributed to suppression of the intramolecular Fe(iii) to Fe(ii) reduction. Attempts to replicate this simple approach to ternary metal sulfides of iron–indium and iron–zinc were unsuccessful, mixtures of binary metal sulfides resulting. Oleylamine is non-innocent in these transformations, and we propose that SSP decomposition occurs *via* primary–secondary backbone amide-exchange with primary dithiocarbamate complexes, [M(S_2_CNHoleyl)_*n*_], being the active decomposition precursors.

## Abstract 2

Heating mixtures of dithiocarbamate complexes in oleylamine leads to the formation of nanoparticulate ternary metal sulfides, the physical nature and phase of which can be tailored depending upon the reaction conditions adopted.

## Graphical abstract

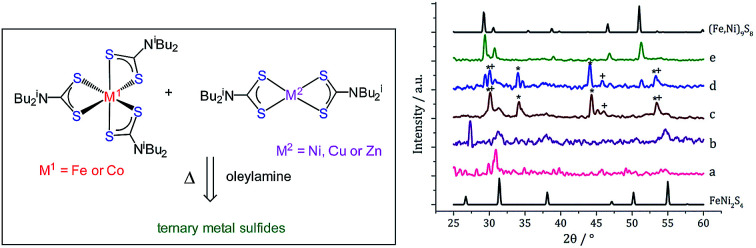

## Introduction

Since the properties of nanomaterials are highly dependent upon their size, shape, composition and architecture, then the development of reproducible and controlled synthetic methodologies presents a major challenge.^1^ Our interest in nanomaterials focuses on the potential role of iron sulfides in prebiotic chemistry, especially the reduction of CO_2_ to biologically useful molecules believed to have occurred at the interface between hydrothermal fluids and the primordial ocean.^2,3^ Thus it has been proposed that iron sulfides found in the chimney cavities of hydrothermal vents^4^ catalysed CO_2_ reduction forming a primitive acetyl-CoA pathway similar to that in contemporary enzymes.^5–7^ Greigite is structurally similar to the Fe_4_S_4_ cluster sub-units found in ferredoxins^8^ and its catalytic nature in CO_2_ activation has been demonstrated,^9,10^ while iron sulfides have also been shown to catalyse CO_2_ reduction.^11^ Thus in a recent communication^9^ we showed that nanoparticles of greigite can reduce CO_2_ under ambient conditions into methanol, formic, acetic and pyruvic acid.

**Chart 1:**
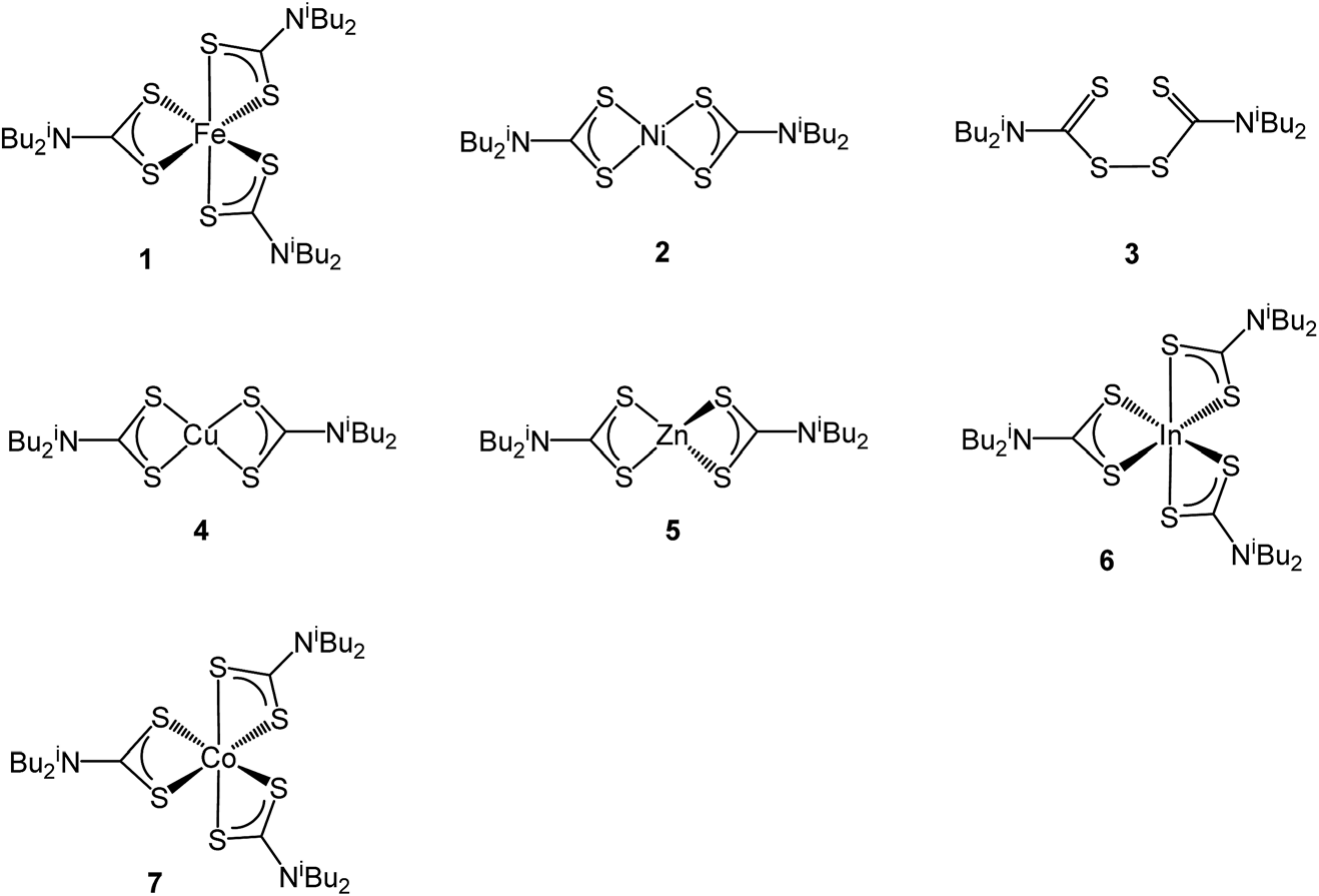


The greigite used for these experiments was prepared from the controlled hydrothermal decomposition of the dithiocarbamate complex [Fe(S_2_CN^i^Bu_2_)_3_] (1) in oleylamine.^12^ Dithiocarbamate complexes^13^ find extensive use as single source precursors (SSPs) towards a range of nanomaterials^14–22^ their utility stemming from their ease of synthesis, and the ability to tune the solubility, volatility and decomposition properties upon making simple changes to the amine substituents. A further advantage of the dithiocarbamate approach to SSPs is the realisation that this simple ligand forms stable complexes with all the transition metals.^12^ Thus in seeking to develop our work on the catalytic properties of metal-sulfide nanomaterials we sought to prepare a range of ternary sulfide nanomaterials, primarily containing iron but also nickel. To do this we have used a range of simple air-stable di-isobutyl-dithiocarbamate complexes (Chart 1) and carried out decomposition studies in oleylamine under a range of conditions. This has successfully led to the synthesis of a number of phases of FeNi, FeCu and NiCo sulfides which are described herein, although attempts to prepare FeZn and FeIn sulfides by this method failed.

## Results and discussion

### (i) Iron–nickel sulfides

Ternary iron-nickel sulfide materials can take a number of phases,^23^ most commonly (Fe,Ni)_9_S_8_ (pentlandite, pentlandite structure) and FeNi_2_S_3_ (violarite, inverse thiospinel structure), although a pyrite type structure (Fe,Ni)S_2_ (bravoite) has also been reported.^24^ There are few examples of the synthesis of nanomaterials of these phases. Bezverkhyy and co-workers synthesised pentlandite nanoparticles *via* a multistep process involving pyrolysis in a H_2_S/N_2_ atmosphere of a poorly-defined precursor synthesised from Fe(SO_4_)·7H_2_O and Ni(SO_4_)·7H_2_O^25^ and Trávníček reported that a nanoparticulate residue of Fe_5_Ni_4_S_8_ resulted when [Ni(S_2_CNBz^i^Pr)_3_][FeCl_4_] was decomposed at 800 °C.^26^ As far as we are aware, there are no reports of either violarite or bravoite being synthesised in the nanoparticle regime.

**Fig. 1:**
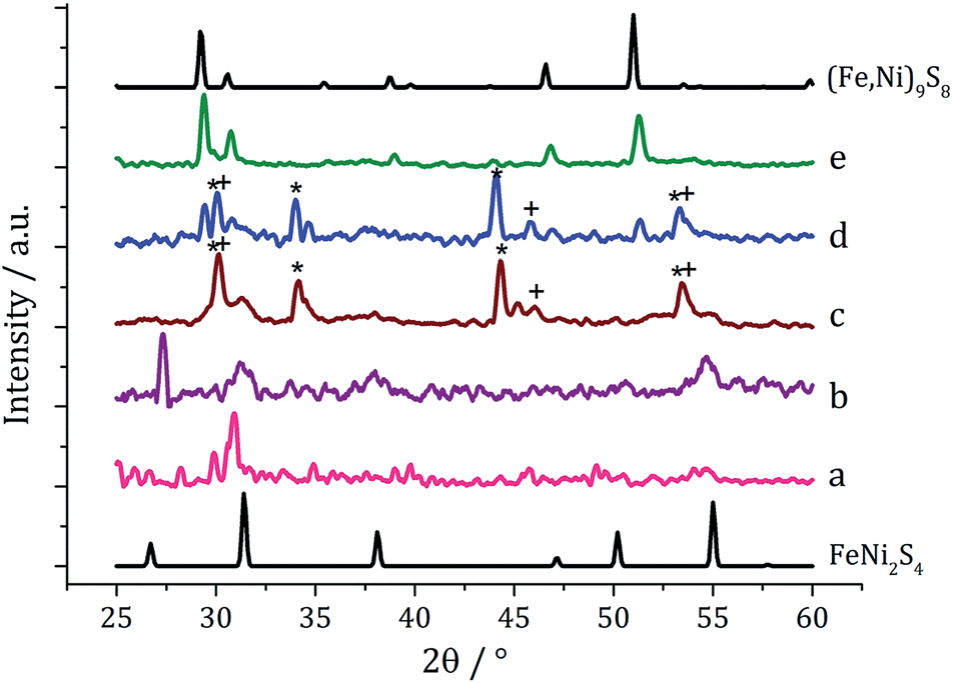
PXRD patterns for samples prepared from 1 with 2 at (a) 150 °C, (b) 180 °C, (c) 230 °C, (d) 260 °C and (e) 280 °C, with reference patterns for FeNi_2_S_4_ (ICDD card no. 47-1740) and (Fe,Ni)_9_S_8_ (ICDD card no. 75-2024). ‘*’ indicates Fe_7_S_8_ peaks and ‘+’ indicates α-NiS peaks.

In our first attempt at preparing ternary iron-nickel sulfide nanomaterials we used [Fe(S_2_CN^i^Bu_2_)_3_] (1) and [Ni(S_2_CN^i^Bu_2_)_2_] (2) as SSPs; decomposition of 2.5 mM quantities of each was carried out in oleylamine at 150, 180, 230, 260 and 280 °C. Upon warming the oleylamine solution a number of temperature-dependent colour changes were observed, being similar to those previously reported in the decomposition of 1 alone, but occurring at *ca.* 10 °C higher.^12^ Thus the initially dark brown solution become suddenly clear and pale yellow at 85 °C, then at 90 °C rapidly went black. Samples were heated to the target temperature and held there for 1 h after which the generated nanoparticles were isolated as black powders and analysed by PXRD (Fig. 1). At 150 °C the material generated is mostly amorphous, but by 180 °C peaks for FeNi_2_S_4_ begin to emerge and at *ca.* 230 °C a mixture of FeNi_2_S_4_, Fe_7_S_8_ and α-NiS is seen. Increasing the temperature to 260 °C sees the emergence of peaks attributed to (Fe,Ni)_9_S_8_, and at 280 °C the crystalline phase of the sample appears to be comprised of pure (Fe,Ni)_9_S_8_. This suggests that FeNi_2_S_4_ (violarite) is metastable, with (Fe,Ni)_9_S_8_ (pentlandite) being thermodynamically favourable.

**Fig. 2:**
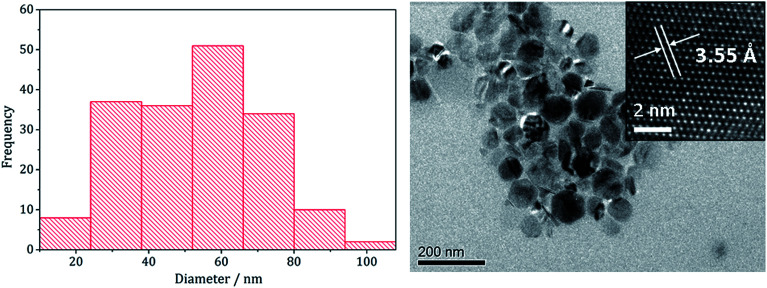
Particle size histogram (left) and TEM image with HRTEM insert (right) of the (Fe,Ni)_9_S_8_ sample prepared from 1 with 2 at 280 °C.

The pure (Fe,Ni)_9_S_8_ produced at 280 °C was analysed by TEM and HRTEM being comprised of roughly hexagonal nanoparticles of 53 nm (SD 18 nm) average diameter (Fig. 2). These are significantly smaller than nanoparticulate pentlandite prepared by Bezverkhyy (Av. 240 nm).^25^ HRTEM analysis reveals a d-spacing of 3.55 Å corresponding to the [220] lattice plane (3.57 Å).

**Fig. 3:**
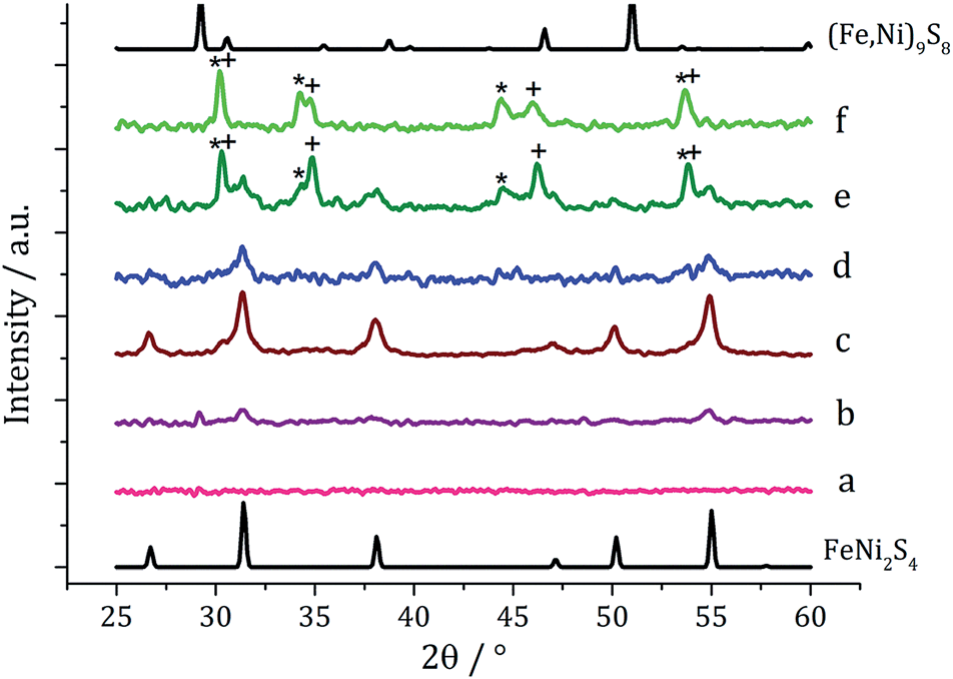
PXRD patterns for samples prepared from mixtures of 1, 2 and 3 at (a) 150 °C, (b) 180 °C, (c) 230 °C, (d) 260 °C, (e) 280 °C, and (f) 300 °C, with reference patterns for FeNi_2_S_4_ (ICDD card no. 47-1740) and (Fe,Ni)_9_S_8_ (ICDD card no. 75-2024). ‘*’ indicates Fe_7_S_8_ peaks and ‘+’ indicates α-NiS peaks.

In recent work we found that decomposition of 1 and 2 alone were significantly influenced by addition of tetra-isobutylthiuram disulfide (3) leading to the increased stability of metastable phases and allowing access to the thiospinel phases Fe_3_S_4_ and Ni_3_S_4_.^22^ For 1 this was related to an intramolecular ligand–metal electron transfer leading to reduction to an Fe(ii) product and extrusion of 3, the addition of which moves the equilibrium towards the Fe(iii) species.^12^ We thus investigated how the addition of 3 would affect the decomposition of mixtures of 1 and 2. Consequently all reactions were repeated with addition of *ca.* 4 equivalents of 3 (*i.e.* 10 mM *ca.* 2 eq. to each precursor). PXRD analysis revealed a similar trend to the samples prepared in the absence of thiuram disulfide but with some notable differences (Fig. 3). Thus, while spectra are similar at low temperatures, at 230 °C seemingly pure FeNi_2_S_4_ is formed, that is without Fe_7_S_8_ and α-NiS impurities seen in the absence of 3. This indicates that addition of 3 stabilises the thiospinel, FeNi_2_S_4_, phase possibly preventing formation of binary metal sulfides. At 260 °C the peaks attributed to FeNi_2_S_4_ broaden and those of Fe_7_S_8_ and α-NiS begin to appear, while at 280 °C the sample is mainly Fe_7_S_8_ and α-NiS, with some FeNi_2_S_4_ remaining.

To probe whether the thermodynamically stable phase, (Fe,Ni)_9_S_8,_ could be accessed in the presence of 3 but at higher temperatures, a decomposition was performed at 300 °C with addition of only 2 eq. of 3. PXRD of the resulting material showed the crystalline phase to be a *ca.* 1 : 1 mix of Fe_7_S_8_ and α-NiS, with neither FeNi_2_S_4_ or (Fe,Ni)_9_S_8_ being present. This suggests addition of 3 prevents formation of (Fe,Ni)_9_S_8_, making binary metal sulfide phases more thermodynamically favourable.

**Fig. 4:**
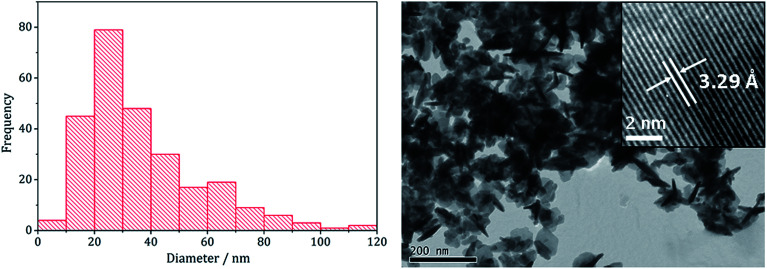
Particle size histogram (left) and TEM image with HRTEM insert (right) of the FeNi_2_S_4_ sample prepared from mixtures of 1–3 at 230 °C.

Analysing the FeNi_2_S_4_ particles produced at 230 °C by TEM (Fig. 4) showed them to be short rods embedded in hexagonal interlocking sheets with an average diameter of 36.5 nm (SD 21 nm), being slightly smaller than (Fe,Ni)_9_S_4_ nanoparticles formed in the absence of 3 (average diameter 53.1 nm). HRTEM analysis of the nanoparticles reveals *d*-spacings of 3.29 Å, corresponding to the [220] lattice plane in FeNi_2_S_4_ (3.35 Å).

**Fig. 5:**
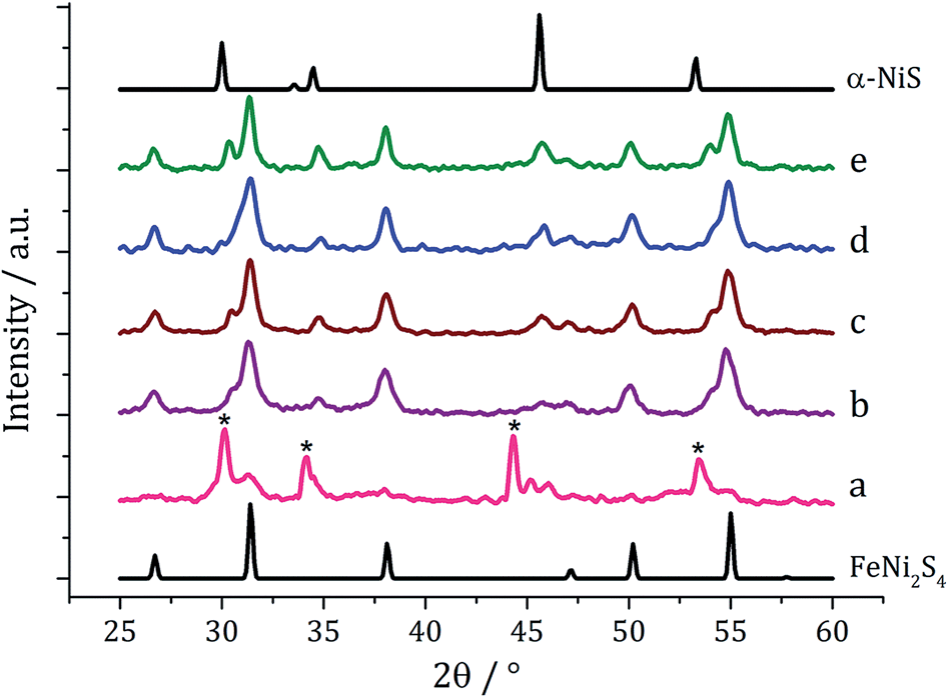
PXRD patterns for samples prepared from 1–2 at concentrations of (a) 2.5 mM, (b) 5 mM, (c) 10 mM, (d) 20 mM and (e) 25 mM, with reference patterns for FeNi_2_S_4_ (ICDD card no. 47-1740) and α-NiS (ICDD card no. 02-1273). ‘*’ indicates peaks for Fe_7_S_8_.

We next varied precursor concentrations, equimolar amounts of 1 and 2 at 2.5, 5, 10, 20 and 25 mM were decomposed in oleylamine at 230 °C. PXRD analysis reveals a trend in the phase of metal sulfide produced with increasing concentration of precursors (Fig. 5). At 2.5 mM the majority of the crystalline sample corresponds to Fe_7_S_8_, with some FeNi_2_S_4_ and α-NiS, while at higher concentrations the crystalline phase is predominantly the thiospinel FeNi_2_S_4_, with some α-NiS impurity. This is consistent with the concentration study performed on 1^12^ where at low precursor concentrations (5 mM) Fe_7_S_8_ was formed, and as the concentration was increased (up to 50 mM) the phase changed to the thiospinel, Fe_3_S_4_. When a similar study was performed on 2, changing concentration had no effect on nickel sulfide phase; α-NiS being formed at all concentrations.

**Fig. 6:**
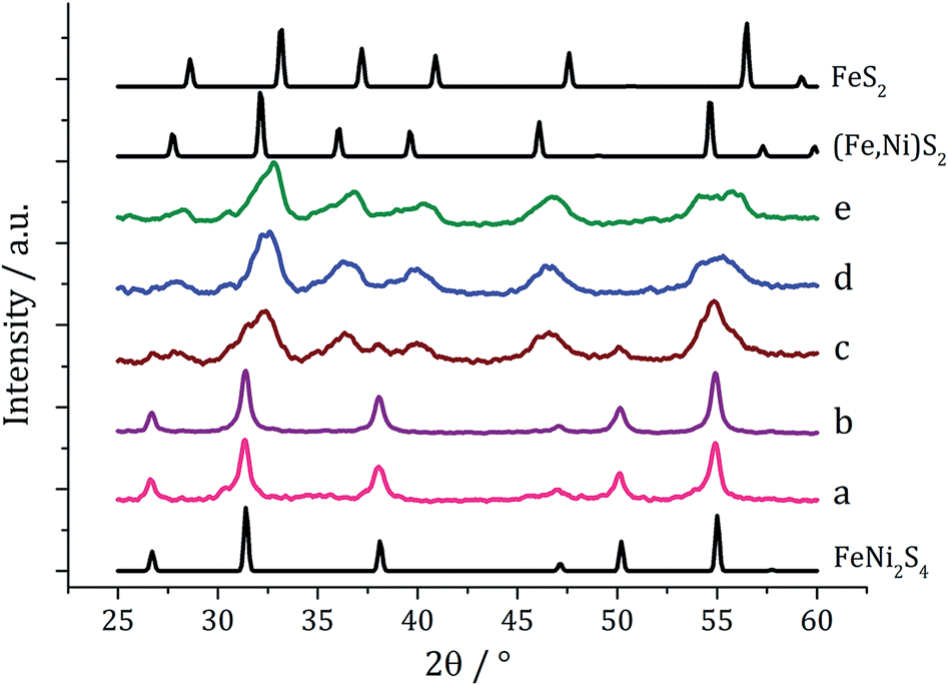
PXRD patterns for samples prepared from mixtures of 1–3 (1 : 1 : 2) at concentrations of 1–2 of (a) 2.5 mM, (b) 5 mM, (c) 10 mM, (d) 20 mM and (e) 25 mM, with reference patterns for FeNi_2_S_4_ (ICDD card no. 47-1740), (Fe,Ni)S_2_ (ICDD card no. 88-1710) and FeS_2_ (ICDD card no. 42-1340).

Experiments were repeated upon addition of four equivalents of 3 and PXRD analysis again shows a concentration-dependent phase change (Fig. 6). At low concentrations pure violarite is formed, but upon increasing the majority of the crystalline material matches the pattern for (Fe,Ni)S_2_ (bravoite, a nickelian pyrite) and for the 1 : 2 : 3 = 20 : 20 : 80 mM sample the pattern matches pure (Fe,Ni)S_2_. Peaks are, broad suggesting that the nanoparticles are small, being consistent with the concentration study on the nickel complex 2 in the presence of 3.^22^ In the ternary system, when the concentration is increased further, additional peaks at slightly higher 2*θ* angles appear, indicative of the presence of FeS_2_ (pyrite). This is interesting since in the binary iron sulfide system, pyrite was not accessible at high concentrations, even after addition of 3. It should also be noted that at higher concentrations, low angle (10–20°) broad peaks are observed, possibly resulting from excess sulfur.

**Fig. 7:**
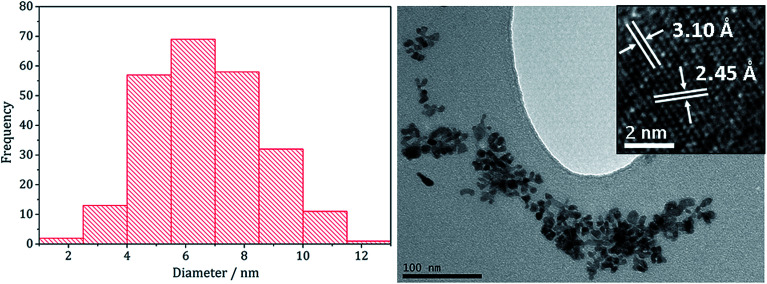
Particle size histogram (left) and TEM image with HRTEM insert (right) of the (Fe,Ni)S_2_ sample prepared from 1–3 at concentrations 20 : 20 : 80 mM.

Nanoparticles produced at 20 mM 1–2 were analysed by TEM, being approximately spherical and with small crystallite size (average diameter 6.7 nm, SD = 1.9 nm), as indicated by the broad peaks in the PXRD pattern. They closely resemble NiS_2_ nanoparticles formed at high concentration of 1 decomposed with 3 (1 : 3 = 20 : 40 mM) which had an average diameter of 4.2 nm (SD = 1.3 nm).^22^ HRTEM analysis (Fig. 7) of the (Fe,Ni)S_2_ nanoparticles reveals *d*-spacings of 3.10 and 2.45 Å, which match the [111] and [200] planes of (Fe,Ni)S_2_ (3.21 and 2.49 Å respectively).

In summary, products of the mixed iron and nickel ternary sulfide system were found to be highly dependent upon both the decomposition temperature and precursor concentration, with similarities to both the iron sulfide and nickel sulfide binary systems. The pentlandite phase (Fe,Ni)_9_S_8_, thiospinel phase FeNi_2_S_4_ and pyrite phase (Fe,Ni)S_2_ could be selectively produced by varying reaction temperature, precursor concentration and employing the tetra-isobutylthiuram disulfide additive 3. We believe that this is the first reported synthesis of the latter two phases in nanoparticulate form.

### (ii) Iron–copper sulfides

Copper–iron sulfide has many known phases including Cu_5_FeS_4_ (bornite), CuFeS_2_ (chalcopyrite) and CuFe_2_S_3_ (cubanite)^27^ and there are numerous reports on their synthesis, although only a few involving the decomposition of dithiocarbamate complexes. Thus, methods to synthesise nanoparticulate copper–iron sulfides include; solvothermal and microwave-assisted decomposition of metal salts with sulfur sources, colloidal synthesis and laser ablation of bulk CuFeS_2_.^28^ Two reports have previously detailed the use of dithiocarbamate SSPs for the synthesis of copper–iron sulfide nanoparticles. Gupta utilised a ‘hot-injection’ method to prepare CuFeS_2_ nanoparticles of *ca.* 12 nm diameter, injecting a mixture of [Cu(S_2_CNEt_2_)_2_] and [Fe(S_2_CNEt_2_)_3_] in oleylamine/dichlorobenzene into a solution of sulfur in oleylamine/trioctylphosphine at 180 °C,^29^ while Pang and co-workers synthesised CuFeS_2_ nanoparticles of *ca.* 6 nm (ref. 30) upon injecting solution of NaS_2_CNEt_2_ in dodecanethiol into a hot (140 °C) solution of FeCl_3_ and CuCl_2_ oleic acid/dodecanethiol; dithiocarbamate complexes presumably being formed *in situ*.^30^

**Fig. 8:**
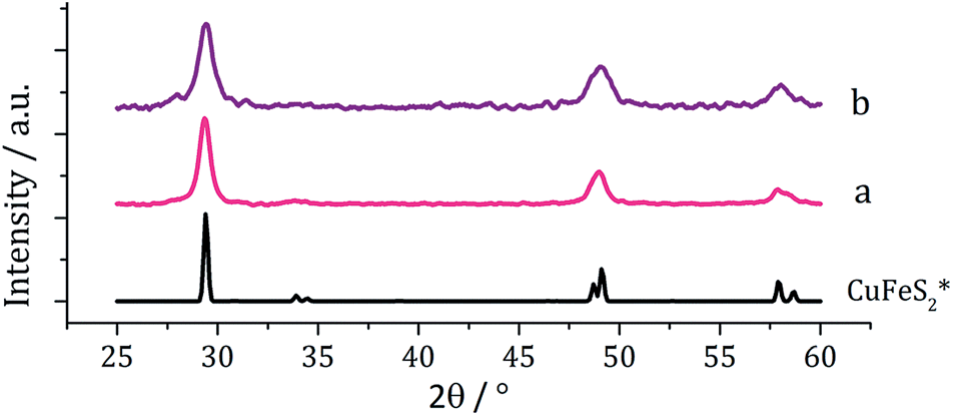
PXRD patterns for samples prepared from (a) 1 and 4 and (b) 1 and 4 with added 3, with reference pattern for bulk CuFeS_2_ (ICDD card no. 37-0471).

**Fig. 9:**
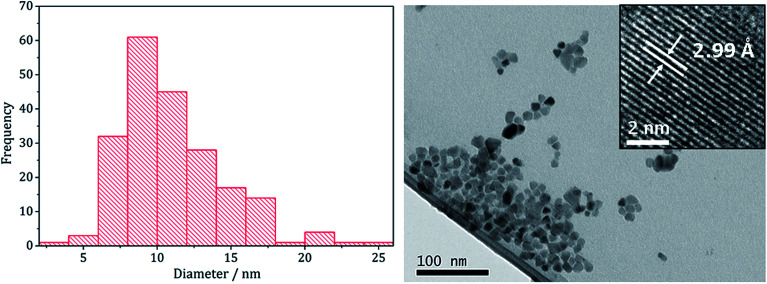
Particle size histogram (left) and TEM image with HRTEM insert (right) of the sample prepared from 1 and 4.

Following on from our methodology described above, we decomposed equimolar amounts (2.5 mM) of brown [Cu(S_2_CN^i^Bu_2_)_2_] (4) and [Fe(S_2_CN^i^Bu_2_)_3_] (1) in oleylamine at 230 °C. The reaction was then repeated with the addition 4 eq. of thiuram disulfide 3, and the resulting brown powders were analysed. No discernible colour changes occurred during these reactions, clear purple dispersions resulting in chlorinated solvents. PXRD analysis (Fig. 8) showed that the crystalline phase produced in both cases corresponded to CuFeS_2_ (chalcopyrite). This is in accordance with previous literature.^29,30^ TEM analysis revealed that addition of 3 had little effect on the size or morphology of the nanoparticles, which were roughly spherical (Fig. 9). Average particle diameters were 11 nm (SD = 4 nm) without 3, and 10 nm (SD = 4 nm) with 3. HRTEM analysis (Fig. 9) revealed a *d*-spacing of 2.99 Å, which matches the [112] plane (3.21 Å, CuFeS_2_).

These results are in accord with those of Gupta and co-workers^29^ who generated similar nanomaterials at 180 °C. Even upon increasing the temperature by 50 °C we saw no significant change in the nanoparticle size suggesting that during nanoparticle formation the duration of burst nucleation and the number of nucleation sites is not affected by temperature.

### (iii) Attempts to prepare iron–zinc sulfides

There are several known phases of the ternary sulfide of iron and zinc; (Zn,Fe)S wurtzite ({ZnFe}S_(W)_, an iron doped wurtzite), (Zn,Fe)S marmatite ({ZnFe}S_(S)_, an iron rich sphalerite) and ZnFe_2_S_4_, a thiospinel^31^ but few reports on nanoparticulate synthesis and none involving a dithiocarbamate SSP. Feng and co-workers prepared thin films of (Zn,Fe)S_(W)_ by MOCVD at 350 °C from [Fe(CO)_5_], Me_2_Zn and H_2_S^32^ and the same material was synthesised in nanoparticulate form upon solvothermal decomposition of [Zn(Aftscz)_2_] and [Zn(AftsczH)_2_Cl_2_] (AftsczH = monoacetylferrocene thiosemicarbazole) in ethylene glycol.^33^ Nanoparticles of (Zn,Fe)S_(S)_ have also been synthesised; Chen *et al.* prepared Zn_0.9_Fe_0.1_S upon annealing nanoparticles produced from a micro-emulsion of [Fe(CO)_5_], [Zn(O_2_CCH_3_)_2_] and CH_3_(CH_2_)_11_OSO_3_Na (sodium dodecyl sulphate) in water with added thioacetamide.^34^

**Fig. 10:**
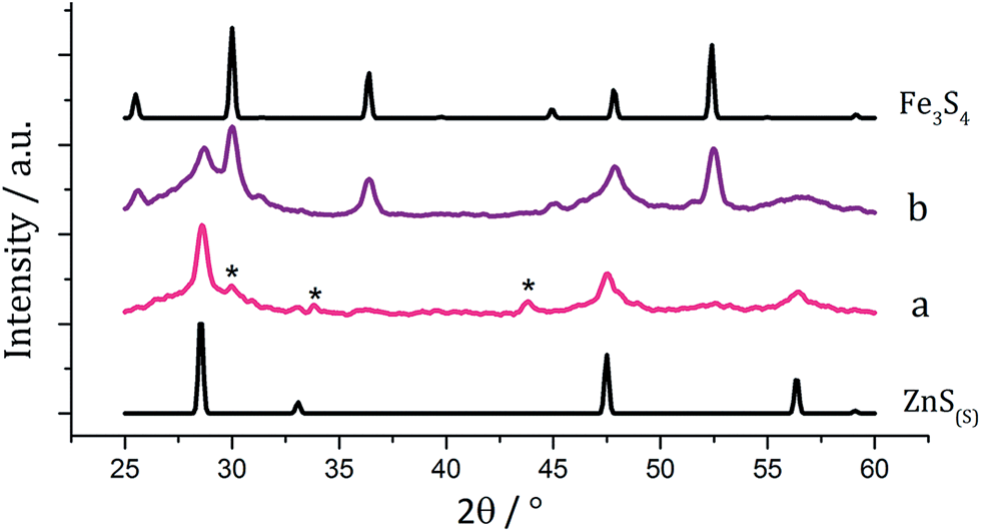
PXRD patterns for samples prepared from (a) 1 with 5 and (b) 1 with 5 and 3, with reference patterns for bulk ZnS_(S)_ (ICDD card no. 77-2100) and Fe_3_S_4_ (ICDD card no. 16-0713). ‘*’ indicates peaks for Fe_7_S_8_.

We used equimolar amounts (2.5 mM) of [Zn(S_2_CN^i^Bu_2_)_2_] (5) and [Fe(S_2_CN^i^Bu_2_)_3_] (1) in oleylamine at 230 °C in the absence and presence of 4 eq. of thiuram disulfide 3. As with previous decompositions involving 1, dark brown solutions suddenly became clear and pale yellow at *ca.* 85 °C, and at 100 °C quickly turned black. Dark brown powders were isolated but PXRD analysis revealed that the crystalline phase corresponded to a mixture of iron sulfide and zinc sulfide phases (Fig. 10). Interestingly, in both the zinc sulfide phase matched to the pattern for the sphalerite rather than the wurtzite phase. Notably the characteristic peak for ZnS_(W)_ at 39.8° 2*θ* is absent. Peaks match well for ZnS_(S)_, and are not shifted to lower angles, as previously observed for mixed iron and zinc sulfide systems^32^ suggesting that no ternary mixed-metal sulfide has been formed. The reason for this is unclear, but possibly the precursors decompose at quite different temperatures, giving rise to separate nucleation events and forming binary metal sulfide nanoparticles. The sample prepared in the absence of 3 appears to also have peaks for the iron sulfide Fe_7_S_8_ and the sample prepared with 3 contains peaks which match well to Fe_3_S_4_, the thiospinel phase of iron sulfide. This is consistent with previous results reported on the iron sulfide binary phase system.

**Fig. 11:**
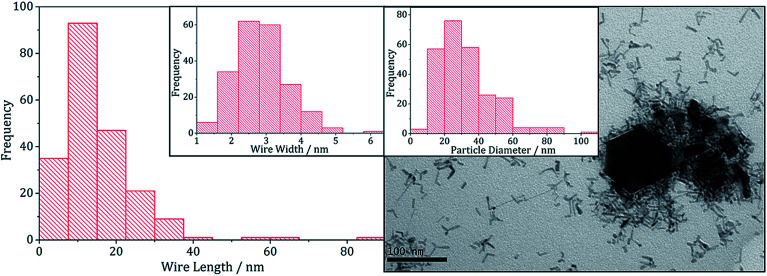
Wire-length histogram with wire-width histogram insert (left) and TEM image with particle diameter histogram insert (right), of the sample prepared from 1 with 5.

TEM images support the PXRD analysis, clearly showing a two phase system (Fig. 11). Samples comprised large hexagonal particles (average diameter 32 nm, SD 16 nm) which resemble the iron sulfide phases observed in the binary system, and thin nanowires (length = 16 nm, SD 15 nm, width = 2.9 nm, SD 0.8 nm) resembling zinc sulfide material produced in the binary system.

### (iv) Attempts to prepare iron–indium sulfides

**Fig. 12:**
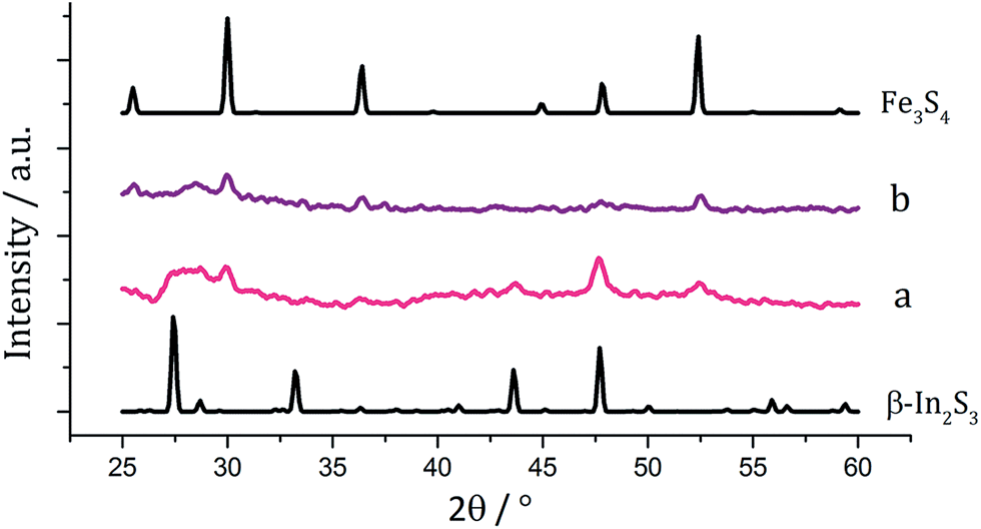
PXRD patterns for samples prepared from (a) 1 with 6, (b) 1 with 6 and 3, with reference patterns for β-In_2_S_3_ (ICDD card no. 25-0390) and Fe_3_S_4_ (ICDD card no. 16-0713).

There have been few reported syntheses of nanoparticulate FeIn_2_S_4_, and none using solvothermal methods. Related to this work, Wu *et al.* recently synthesised FeIn_2_S_4_ microspheres using an indium dithiocarbamate precursor.^35^ Equimolar amounts (2.5 mM) of pale yellow [In(S_2_CN^i^Bu_2_)_3_] (6) and dark brown [Fe(S_2_CN^i^Bu_2_)_3_] (3) were decomposed in oleylamine, being repeated upon addition of 3, and the resulting brown powders were analysed. PXRD patterns (Fig. 12) did not match that of the mixed iron indium sulfide, FeIn_2_S_4_, being broad and indicative of low crystallinity. Peaks for Fe_3_S_4_ were detectable in both, as well as for β-In_2_S_3_ in the sample prepared with added 3. As with the iron-zinc sulfide chemistry ternary mixed-metal sulfides were absent. Interestingly, unlike the iron zinc sulfide samples, peaks for pyrrhotite are not observed in the sample prepared without 3, but peaks matching Fe_3_S_4_ are seen in both. This might suggest that the presence of the indium precursor 6 or indium sulfide product β-In_2_S_3_ helps to stabilise the iron sulfide thiospinel.

### (v) Nickel–cobalt sulfides

Although the focus of our work with ternary chalcogenides is on mixed-metal iron sulfides, many other combinations of mixed-metal sulfide materials are possible. The nickel–cobalt sulfide system has several possible phases including; NiCo_8_S_8_ (pentlandite structure), NiCo_2_S_4_ and CoNi_2_Co_4_ (both thiospinel structure). Nanoparticles of NiCo_8_S_8_ have been synthesised by Bezverkhyy *et al.* in a two-step process; firstly Co(NO_3_)_2_ and Ni(NO_3_)_2_ were reacted with Na_2_S in water and then the dried precipitate was heated with H_2_S/H_2_ for 3 h at 300 °C.^25^ Urchin-like nanostructures of NiCo_2_S_4_ were synthesised by Jiang and co-workers in a two-step process involving the hydrothermal decomposition of NiCl_2_ and CoCl_2_ with urea in an autoclave at 140 °C, followed by a further reaction with Na_2_S at 160 °C (ref. 36) and nanoparticles of CoNi_2_S_4_ were formed in an autoclave by Pang *et al.*, from the slow (24 h) decomposition of [Co(O_2_CMe)_2_] and [Ni(O_2_CMe)_2_] mixtures with sulfur dissolved in an oleylamine/anisole mixture.^37^

**Fig. 13:**
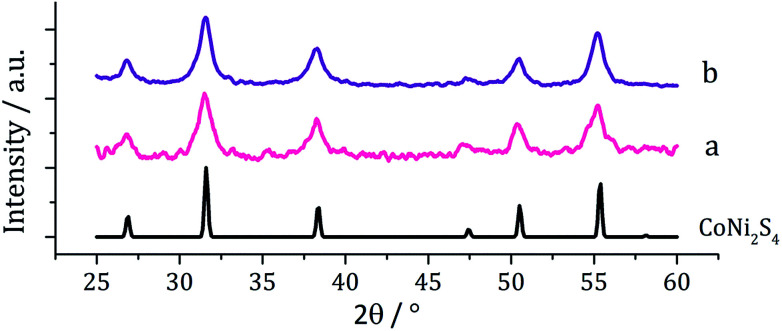
PXRD patterns for samples prepared from (a) 2 with 7 and (b) 2 with 7 and 3, with reference pattern for bulk CoNi_2_S_4_ (ICDD card no. 22-0334).

Equimolar amounts (2.5 mM) of [Ni(S_2_CN^i^Bu_2_)_2_] (2) and [Co(S_2_CN^i^Bu_2_)_3_] (7) were decomposed in oleylamine at 230 °C, being repeated upon addition of thiuram disulfide 3, and the resulting brown powders analysed. PXRD analysis (Fig. 13) revealed no significant shift in peaks between the two samples, indicating that 3 has no observable effect on the phase of ternary sulfide produced. In addition, to elucidate whether the nickel or cobalt rich thiospinel structures had been formed (CoNi_2_S_4_ or NiCo_2_S_4_ respectively) both samples were analysed by energy-dispersive X-ray spectroscopy (EDX) and found to have atomic compositions of cobalt, nickel and sulfur of 1.76 : 1.60 : 4 and 1.52 : 1.32 : 4, respectively. Although the ratios of metal to sulfur vary slightly, they remain approximately 1.1 : 1 Co : Ni, indicating an intermediate (Ni,Co)_3_S_4_ phase. The difference between the PXRD patterns for CoNi_2_S_4_ and NiCo_2_S_4_ is small; nickel and cobalt having very similar ionic radii and electronegativity, and the patterns obtained for the samples synthesised here closely match both.

**Fig. 14:**
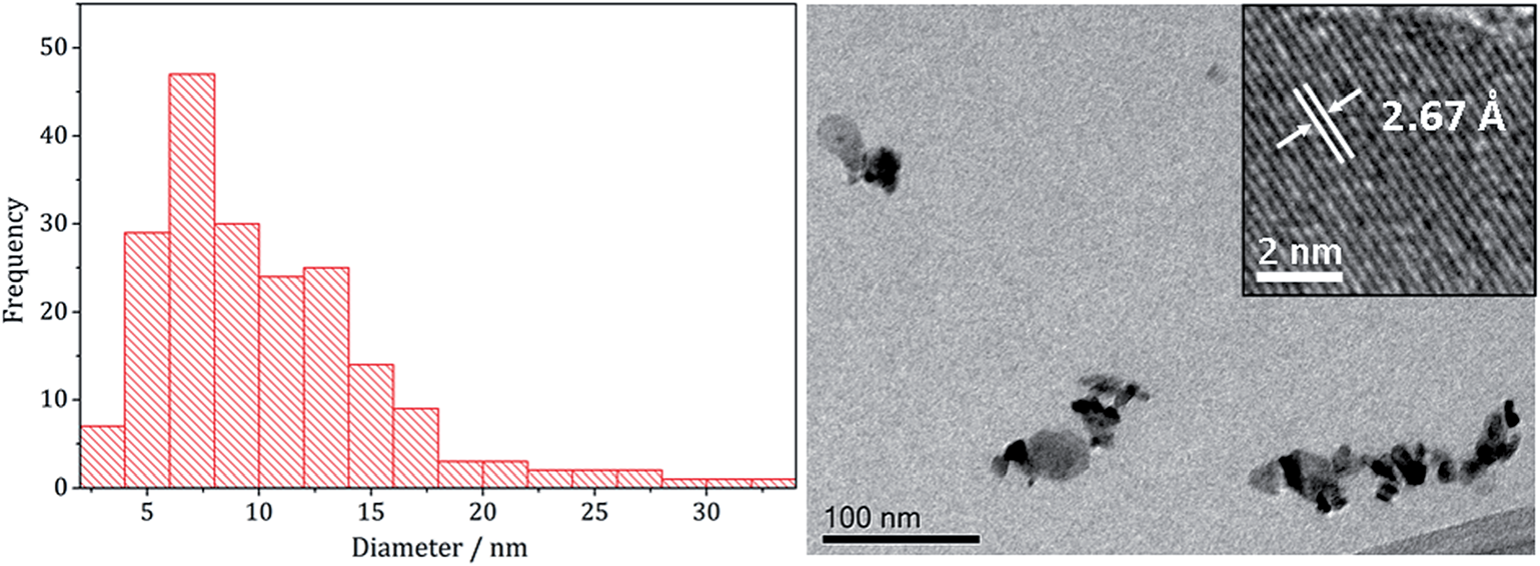
Particle size histogram (left) and TEM image with HRTEM insert (right) of the (Ni,Co)_3_S_4_ sample prepared from 2 with 7 and 3.

TEM analysis revealed the addition 3 also had little effect on the size or morphology of the nanoparticles formed, which were roughly spherical (Fig. 14). The average particle diameters were 10 nm (SD = 5 nm) for the sample prepared without 3, and 10 nm (SD = 4 nm) with 3. Nanoparticles of CoNi_2_S_4_ previously reported by Pang *et al.*^37^ were reported to have ‘quasi-spherical’ morphology with an average diameter of approximately 8–15 nm, similar to those produced in the current study. HRTEM analysis (Fig. 13) of the current (Ni,Co)_3_S_4_ nanoparticles reveals a *d*-spacing of 2.67 Å, which matches the [222] plane (2.72 Å, CoNi_2_S_4_).

## Summary and conclusions

Nanomaterials of iron–nickel sulfide, iron–copper sulfide and nickel–cobalt sulfide were successfully prepared *via* the decomposition of mixtures of simple metal–dithiocarbamate complexes in oleylamine, and for iron–nickel mixtures, various sulfide phases were accessible upon varying decomposition conditions. In contrast, attempts to synthesise iron–zinc sulfide and iron–indium sulfide *via* this method were unsuccessful, producing only binary metal sulfides. This may be due to the precursor complexes decomposing at different temperatures, although this has not been fully established. We have previously shown that [Fe(S_2_CN^i^Bu_2_)_3_] (1) undergoes an intramolecular electron-transfer process at *ca.* 80 °C in oleylamine to generate [Fe(S_2_CN^i^Bu_2_)_2_] and thiuram disulfide (S_2_CN^i^Bu_2_)_2_ (3).^12^ This also occurs during decompositions carried out here as shown by the sudden colour change from brown to yellow at *ca.* 80–90 °C. Thus the decomposition mixtures contain an Fe(ii) and not an Fe(iii) dithiocarbamate complex. Consistent with this supposition, addition of 3 to the decomposition mixture has a significant effect on the precise nature of the nanomaterials formed. We have also previously shown that decomposition of [Ni(S_2_CN^i^Bu_2_)_2_] (2) in oleylamine occurs *via* an initial amide-exchange process to generate the primary amine derivative [Ni(S_2_CNHoleyl)_2_]^21,22^ and we have evidence that [Fe(S_2_CN^i^Bu_2_)_2_] behaves in the same way.^12^ Thus, while it appears that iron–nickel sulfides result from decomposition of [Fe(S_2_CN^i^Bu_2_)_3_] (1) and [Ni(S_2_CN^i^Bu_2_)_2_] (2), most likely it is [Ni(S_2_CNHoleyl)_2_] and [Fe(S_2_CNHoleyl)_2_] that are actually decomposing *via* extrusion of oleylNCS. We have less information about the precise nature of 4–7 in oleylamine but cannot rule out similar amide-exchange reactions being important. Hence, a general comment about the decomposition of dithiocarbamate SSPs in oleylamine (and other amines) is that, while changes to the alkyl-substituents may appear to lead to different nanomaterial sizes and shapes, this is most likely related to the rate of the amide-exchange reaction rather than the relative strengths of the C–S bonds that are cleaved. This may account for the failed attempts to prepare iron–zinc and iron–indium sulfides, in which we obtained only mixtures of binary sulfides. Thus if amide-exchange (and subsequent decomposition) is much faster for [Fe(S_2_CN^i^Bu_2_)_2_] than either [Zn(S_2_CN^i^Bu_2_)_2_] or [In(S_2_CN^i^Bu_2_)_3_] then it is easy to see how iron–sulfides would be generated first followed by a slower process to afford zinc or indium sulfide. Consequently we are focusing our current efforts on the synthesis (and subsequent decomposition) of primary-amine dithiocarbamate derivatives, [M(S_2_CNHR)_*n*_], since this should allow for far better control over the decomposition process (*e.g.* lower temperatures, non-amine solvents *etc.*) potentially providing access to a wider range of binary and ternary metal sulfide nanomaterials.

## Experimental section

### General procedures

Unless otherwise stated, manipulations were performed under a dry, oxygen-free dinitrogen atmosphere using standard Schlenk techniques or in a MBRAUN Unilab glovebox. All solvents used were stored in alumina columns and dried with anhydrous engineering equipment, such that the water concentration was 5–10 ppm. All other reagents were procured commercially from Aldrich and used without further purification.

### Physical measurements

^1^H and ^13^C{^1^H} NMR spectra were obtained on either a Bruker Avance III 400 or Avance 600 spectrometers. All spectra were recorded using CDCl_3_ which was dried and degassed over molecular sieves prior to use; ^1^H and ^13^C{^1^H} chemical shifts are reported relative to SiMe_4_. Mass spectra were obtained using either Micromass 70-SE spectrometer using Electron Ionisation (EI) or a Thermo Finnigan MAT900xp spectrometer using Fast Atom Bombardment (FAB) ionisation. Elemental analysis was carried using Elemental Analyser (CE-440) (Exeter Analytical Inc). PXRD were measured on a Bruker AXS D4 diffractometer using CuKα_1_ radiation, patterns obtained being compared to database standards. For TEM characterisation a 4 μl droplet of nanoparticle suspension (CHCl_3_) was placed on a holey carbon-coated copper TEM grid and allowed to evaporate in air under ambient laboratory conditions for several minutes. TEM images were obtained using a JEOL-1010 microscope at 100 kV equipped with a Gatan digital camera. HRTEM measurements were collected using a Jeol 2100 (high resolution) TEM with a LaB_6_ source operating at an acceleration voltage of 200 kv. Micrographs were taken on a Gatan Orius Charge-coupled device (CCD).

### Synthesis and characterisation of molecular precursors

#### Na[S_2_CN^i^Bu_2_]^22^

^i^Bu_2_NH (5.24 mL, 30 mmol) was added to NaOH (1.20 g, 30 mmol) in water (50 mL). To this mixture CS_2_ (1.80 mL, 30 mmol) was added dropwise over 10 min and the mixture stirred for 1 h. The dithiocarbamate salt was then used *in situ* adding various equivalents to the respective metal salts being dependent upon the stoichiometry of the product.

#### [Fe(S_2_CN^i^Bu_2_)_3_] (1)^38^

A solution of FeCl_3_ (1.62 g, 10 mmol) in water (50 mL) was added dropwise over 5 min, whereupon a black precipitate formed. This mixture was vigorously stirred for 2 h, filtered, washed with water (3 × 30 mL) and evaporated to dryness. The resulting black powder was dissolved in 100 mL of CH_2_Cl_2_ and stirred with magnesium sulphate for 30 min, after which the mixture was filtered and the filtrate dried. Yield 5.55 g, 83%. Anal. Calc. for C_27_H_54_N_3_S_6_Fe: C, 48.48; H, 8.14; N, 6.23. Found: C, 48.52; H, 8.26; N, 6.23. MS: *m*/*z* 669 [M^+^], 464 [M^+^ − C_9_H_18_NS_2_]. IR (*ν*_max_ cm^−1^): 1482 (s) [N=C], 992 (s), 1244 (s) [C=S], 1145 (s) [C_2_N].

#### [Ni(S_2_CN^i^Bu_2_)_2_] (2)^22^

A solution of NiCl_2_·6H_2_O (2.38 g, 10 mmol) in water (50 mL) was added dropwise over 5 min, whereupon a green precipitate formed. This mixture was vigorously stirred for 2 h, filtered, washed with water (3 × 30 mL) and evaporated to dryness. The resulting green powder was dissolved in 100 mL of CH_2_Cl_2_ and stirred with magnesium sulphate for 30 min, after which the mixture was filtered and the filtrate dried. Yield 3.97 g, 85%. Anal. Calc. for C_18_H_36_N_2_S_4_Ni: C, 46.25; H, 7.76; N, 5.99. Found: C, 46.23; H, 7.81; N, 6.03. ^1^H NMR *δ*/ppm (CDCl_3_): 0.91 (d, *J* = 6.6 Hz, 24H, C*H*_3_), 2.17 (m, *J* = 6.8 Hz, 4H, C*H*), 3.40 (d, *J* = 7.7 Hz, 8H, C*H*_2_). ^13^C{^1^H} NMR *δ*/ppm (CDCl_3_): 20.1 (*C*H_3_), 27.0 (CH), 56.3 (*C*H_2_), 208.4 (*C*S_2_). MS: *m*/*z* 467 [M^+^], 171 [S_2_CN^I^Bu_2_]. IR (*ν*_max_ cm^−1^): 1508 (s) [N=C], 981 (s) [C=S].

#### (S_2_CN^i^Bu_2_)_2_ (3)^22^

^i^Bu_2_NH (2.62 mL, 15 mmol) was added to NaOH (0.60 g, 15 mmol) in water (50 mL). To this mixture CS_2_ (0.90 mL, 15 mmol) was added dropwise over 10 min and the mixture stirred for 1 h. An aqueous solution (20 mL) of K_3_[Fe(CN)_6_] (4.94 g, 15 mmol) was added dropwise over 10 min and stirred vigorously for 2 h. The solution was filtered using a Büchner funnel, washed with water (1 × 20 mL) and dried. The resulting beige solid was crushed to a powder using a mortar and pestle, washed with water (3 × 30 mL) and dried to produce a white powder. Yield 2.79 g, 91%. ^1^H NMR *δ*/ppm (CDCl_3_): 0.93 (d, *J* = 6.3 Hz, 12H, C*H*_3_), 1.04 (d, *J* = 6.3 Hz, 12H, C*H*_3_), 2.49 (m, 4H, C*H*), 3.84 (m, 8H, C*H*_2_). ^13^C{^1^H} NMR *δ*/ppm (CDCl_3_): 20.3 (*C*H_3_), 20.5 (*C*H_3_), 26.1 (*C*H), 28.7 (*C*H), 61.8 (*C*H_2_), 65.5 (*C*H_2_), 194.3 (*C*S_2_). Anal. Calc. for C_18_H_36_N_2_S_4_: C, 52.89; H, 8.88; N, 6.85. Found: C, 52.73; H, 9.07; N, 6.90. MS: *m*/*z* 408 [M^+^], 204 [M^+^ − (S_2_CN^I^Bu_2_)], 172 [M^+^ − (SCN^I^Bu_2_)].

#### [Cu(S_2_CN^i^Bu_2_)_2_] (4)^39^

A solution of CuCl_2_ (0.67 g, 5 mmol) in MeOH (50 mL) was added dropwise over 5 min, whereupon a black precipitate formed. This mixture was vigorously stirred for 4 h, filtered, and then washed and dried, yielding a black powder, 2.36 g, 75%. Anal. Calc. for C_18_H_36_N_2_S_4_Cu: C, 45.78; H, 7.68; N, 5.93. Found: C, 45.59; H, 7.71; N, 6.20. MS: *m*/*z* 471 [M^+^], 172 [SCN^i^Bu_2_].

#### [Zn(S_2_CN^i^Bu_2_)_2_] (5)^40^

A solution of ZnSO_4_·7H_2_O (1.44 g, 5.0 mmol) in water (50 ml) was added dropwise over 5 min, whereupon a white precipitate formed. This mixture was vigorously stirred for 4 h, filtered, and then washed and dried, yielding an off-white powder, 2.09 g, 88%. Anal. Calc. for C_18_H_36_N_2_S_4_Zn: C, 45.60; H, 7.65; N, 5.91. Found: C, 46.61; H, 7.97; N, 5.59. ^1^H NMR *δ*/ppm (CDCl_3_): 0.97 (d, *J* = 6.6 Hz, 24H, C*H*_3_), 2.39 (m, *J* = 6.8 Hz, 4H, C*H*), 3.70 (d, *J* = 7.5 Hz, 8H, C*H*_2_). ^13^C{^1^H} NMR *δ*/ppm (CDCl_3_): 20.3 (*C*H_3_), 27.1 (*C*H), 62.2 (*C*H_2_), 204.5 (*C*S_2_). MS: *m*/*z* 472 [M^+^], 268 [M^+^ − S_2_CN^i^Bu_2_], 383 [ZnS(SCN^i^Bu_2_)_2_ − ^i^Bu].

#### [In(S_2_CN^i^Bu_2_)_3_] (6)^41^

A solution of InCl_3_ (1.11 g, 5 mmol) in water (50 ml) was added dropwise over 5 min, whereupon a white precipitate formed. This mixture was vigorously stirred for 4 h, filtered, and then washed and dried, yielding a white powder. Large colourless crystals were obtained by slow evaporation from CH_2_Cl_2_. Yield 3.28 g, 90%. Anal. Calc. for C_27_H_54_N_3_S_6_In: C, 44.55; H, 7.48; N, 5.77. Found: C, 44.59; H, 7.53; N, 5.54. ^1^H NMR *δ*/ppm (CDCl_3_): 0.95 (d, *J* = 6.6 Hz, 24H, C*H*_3_), 2.41 (m, *J* = 6.8 Hz, 4H, C*H*), 3.63 (d, *J* = 7.5 Hz, 8H, C*H*_2_). ^13^C{^1^H} NMR *δ*/ppm (CDCl_3_): 20.5 (*C*H_3_), 27.1 (*C*H), 64.0 (*C*H_2_), 203.6 (*C*S_2_). MS: *m*/*z* 670 [M^+^ − ^i^Bu], 523 [M^+^ − S_2_CN^i^Bu_2_], 467 [In(S_2_CN^i^Bu_2_)_2_ − ^i^Bu].

#### [Co(S_2_CN^i^Bu_2_)_3_] (7)^42^

A solution of CoCl_2_·6H_2_O (1.00 g, 4.20 mmol) in water (100 ml) was added dropwise over 5 min, whereupon a dark green precipitate formed. This mixture was vigorously stirred for 2 h, filtered, washed with water (3 × 30 mL) and evaporated to dryness. The resulting green powder was dissolved in 100 mL of CH_2_Cl_2_ and stirred with magnesium sulphate for 30 min, after which the mixture was filtered and the filtrate dried *in vacuo*. Yield 1.83 g, 65%. Anal. Calc. for C_27_H_54_N_3_S_6_Co: C, 48.25; H, 8.10; N, 6.25. Found: C, 48.17; H, 8.16; N, 6.46. MS: *m*/*z* 671 [M^+^], 467 [M^+^ − S_2_CN^i^Bu_2_].

### Decomposition studies

In a typical synthesis the dithiocarbamate complexes (2.5 mM) were added to oleylamine (20 mL) in a three-neck round bottom flask attached to a water condenser and evacuated and refilled with nitrogen repeatedly for *ca.* 15 minutes. The solution was heated to 230 °C for 1 h. The mixture was slowly cooled to room temperature, whereupon methanol (80 mL) was added with stirring. The mixture was centrifuged and the solution decanted leaving behind the resultant nanoparticles. This procedure was repeated three times and then the material was dried under vacuum.

## Conflicts of interest

There are no conflicts of interest to declare.

## Supplementary Material
